# Spinal-Pelvic Dissociation in Pregnancy: Surgical Fixation of Culture-Negative Extrapulmonary Tuberculosis

**DOI:** 10.1155/2020/9769076

**Published:** 2020-04-10

**Authors:** Ali Taha, Jessica Rogers, Timothy Bishop, Darren F. Lui, Madhusree Ghosh, Paul Gillespie

**Affiliations:** St. George's Hospital, University of London, UK

## Abstract

A 33-year-old gravid female from Pakistan presented to the Emergency Department with persistent, intractable low back pain and neuropathic left L5 leg pain, associated with a left foot drop. There was a notable history of weight loss for 1 year. Investigations revealed a large collection in the right posterior paraspinal muscles tracking from a large bony defect in the right half of her sacrum extending into the pelvis. The collection was suggestive of an abscess and underwent US-guided aspiration. Culture, PCR examination, and bone biopsy were culture-negative for tuberculosis (TB). Samples taken from the placenta showed two small granulomata in the chorionic villi only. A multidisciplinary approach commenced with initiation of empirical TB treatment and attempted normal vaginal delivery. An urgent caesarean section for the delivery of the baby was required for failure to proceed. Spinal-pelvic stabilization in two stages was performed for the unstable fracture pattern, followed by pharmacotherapy and physiotherapy rehabilitation. At 12-month follow-up, the patient showed resolving TB and eradication of the paraspinal abscess. There was bony union and stability of the spinal-pelvic reconstruction. Back pain and sciatica can be common in pregnancy. However, this case highlights a rare occurrence of culture-negative extrapulmonary TB leading to an unstable spinal-pelvic fracture requiring a multidisciplinary approach for careful obstetric and orthopaedic treatment with empirical treatment by the infectious disease team and microbiology.

## 1. Background

Tuberculosis (TB) was declared a public health emergency by WHO in 2005. The disease is a significant contributor to maternal mortality and is among the three leading causes of death among women aged 15–45 years in highly populated areas of the world. The exact incidence of tuberculosis in pregnancy is estimated to be similar to that of the general population [1]. TB prevalence in England has been declining yearly from 5,070 TB diagnoses in 2017 to 4,655 in 2018 with an estimated 8.2% decline [2]. London is recognised as the capital of TB in Europe compared to other European countries [3]. In developed nations, most cases of spinal tuberculosis are seen primarily in immigrants from endemic countries. Approximately 10% of patients with extrapulmonary tuberculosis have skeletal involvement. The spine is the most common skeletal site affected, followed by the hip and knee. Spinal tuberculosis accounts for almost 50% of cases of extrapulmonary skeletal tuberculosis [4, 5].

Relying on investigations to detect TB can be a diagnostic challenge for physicians. 23% of reported TB cases were culture-negative in the United States in 2013 [6]. The increasing number of culture-negative TB patients is a serious concern as patients may be easily misdiagnosed, delaying treatment, leading to increased morbidity and mortality, and encouraging further transmission.

Diagnosis of spinal tuberculosis in pregnancy is a challenging issue for clinicians. The progression of spinal tuberculosis is slow and insidious. The total duration of the illness varies from a few months to a few years, with average disease duration ranging from 4 to 11 months. Usually, patients seek advice only when there is severe pain, marked deformity, or neurological symptoms [4]. Moreover, the symptoms may initially be ascribed to the pregnancy, and the normal weight gain in pregnancy may temporarily mask the associated weight loss. Obstetric complications of TB include spontaneous abortion, small for gestational age fetus, preterm labour, low birth weight, and increased neonatal mortality. Congenital TB, though rare, is associated with high perinatal mortality [1].

Tuberculosis of the spine, with lumbopelvic osseous destruction, complicated with neurological involvement in the presence of gravid uterus in the third trimester, have rarely been reported in the literature. None have reported gross instability requiring instrumentation and fixation following urgent caesarean section. This is the first case report of an open reduction and internal fixation of pelvic fractures with concomitant posterior instrumentation for spinal-pelvic dissociation due to extensive infiltration of culture-negative extrapulmonary skeletal tuberculosis disease.

## 2. Case Presentation

A 33-year-old woman, originally from Pakistan, immigrated to the United Kingdom in 2014. She was a nonsmoker and presented at 37 weeks of pregnancy in the Emergency Department with persistent back pain, paraesthesiae of the left L5 dermatome, and numbness in the S1-2 dermatomes. Examination revealed bilateral suprasacral masses with no signs of contusion or trauma. Motor examination showed MRC 4/5 hip flexion (L2), 4/5 knee extension (L3), 2/5 ankle (L4), EHL dorsiflexion (L5), and absent ankle reflexes (S1, 2). Neurological examination of the cranial nerves, bilateral upper limbs, and right lower limb was unremarkable. PR revealed normal voluntary anal contraction and perianal sensation, with normal bladder and bowel functions. Chest examination was clear, with equal air entry and normal respiratory rate, and no cervical, axillary lymphadenopathy.

She was discharged but returned again at 40 weeks gestation with intractable back pain, left L5 sciatica, and left foot drop. Further history revealed no other constitutional symptoms except weight loss which had been notable over the period of one year. Obstetric examination of the foetus showed small for gestational age (SGA) and symphysis fundal height measuring 33 weeks of gestational age in 40 weeks of pregnancy.

## 3. Investigations

**Figure 1:**
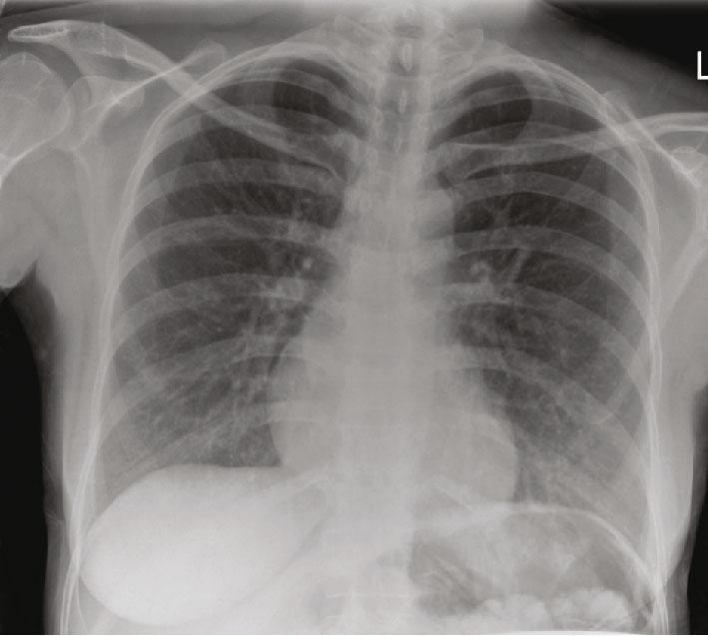
Chest X-ray showing mild bronchial thickening and minimal linear scarring in the left lung base but no definitive focal consolidation, Ghon's focus, or lymphadenopathy.

Due to a high level of suspicion for TB, a CXR was requested after clinical examination. It showed mild bronchial thickening and minimal linear scarring in the left lung base but no definitive focal consolidation, Ghon's focus, or lymphadenopathy (Figure 1).

**Figure 2:**
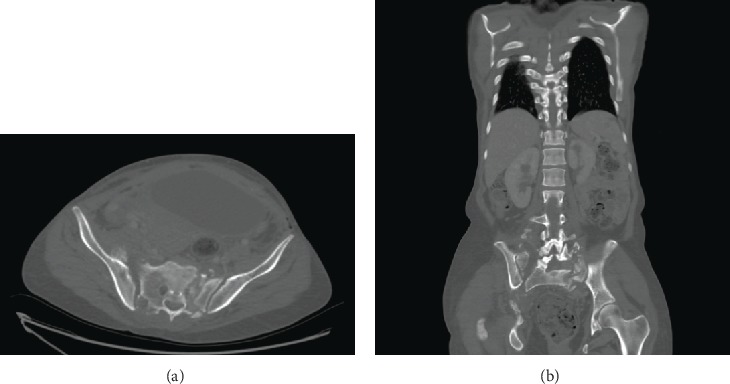
(a, b) CT scan of the pelvis and spine in axial (a) and coronal (b) cuts, respectively, showing spinopelvic dissociation with severe osseous destruction extending from the ala into S1 and S2.

A CT scan of the pelvis and the spine was obtained showing spinopelvic dissociation with osseous destruction extending into S1 and S2 (Figure 2).

**Figure 3:**
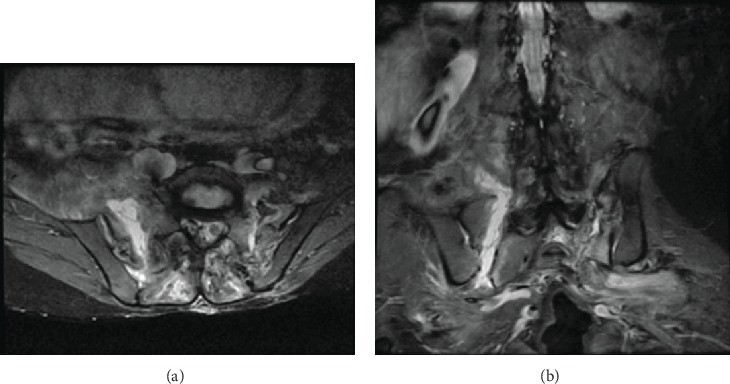
MRI showed an extensive oedema of the posterior paraspinal muscles bilaterally from L4 to S2 levels and diffuse marrow oedema of the sacrum. A large collection in the right posterior paraspinal muscles tracking inferiorly and anteriorly through a large bony defect in the right half of the sacrum seen in the axial (a) and coronal (b) views. There are bilateral spinal-pelvic fractures and dissociation.

MRI showed extensive oedema of the posterior paraspinal muscles bilaterally from L4 to S2 levels and diffuse marrow oedema of the sacrum. A large collection in the right posterior paraspinal muscles tracked inferiorly and anteriorly through a large bony defect in the right half of the sacrum (Figure 3) which extends anteriorly into the pelvic sidewall just posterior to the iliopsoas muscle.

The collection appears hypointense on T1, hyperintense on T2, and STIR with multiple internal septae and a thick wall suggestive of an abscess, measuring 4.8 × 1.6 cm in axial dimensions and 11.1 cm superoinferiorly. A similar small collection was seen on the left side coursing through a linear defect in the left half of the sacrum. It measured 3.5 × 0.5 cm in axial dimensions, which courses into the greater sciatic notch causing mass effect on the lumbosacral plexus. A few more tiny loculations are also seen medially in the right iliacus muscle and marrow oedema in the pedicles of the L5 vertebra.

Vertebral discs were spared (good discs); however, there were disc bulges from the L2-3 to L5-S1 levels. The L3-4 and L4-5 disc bulges contact the traversing L4 and L5 nerve roots bilaterally. Dehydration signals change in the L2-3 and L4-5 discs. The cord returns to normal signal intensity.

### 3.1. Invasive Investigations

This was followed by paraspinal abscess US-guided drainage, which showed mixed echogenicity collection with multiple locules seen in the right sacroiliac joint space.

A bone biopsy was performed. Bone and soft tissue samples obtained were sent to microbiology for microscopy, culture, and sensitivity (MCS), TB, and prolonged cultures. Microbiology sample revealed that acid-fast bacilli were not detected, and TB culture bottles revealed no growth after 42 days.

PCR TB was done, and the molecular amplification test was negative for *Mycobacterium tuberculosis* complex.

A sample from the placenta detected two small granulomata within the chorionic villi. These samples were taken after delivery but notably after the TB treatment was initiated.

## 4. Treatment

The patient was assessed by the infectious diseases team and commenced on an empirical antituberculous treatment protocol of pyridoxine 10 mg OD, rifampcin/isoniazid INH 300/150 mg OD, and ethambutol 800 mg one day after the admission. It was decided by the MDT that an attempt at normal delivery could be safely attempted.

At 40 + 2/40 weeks gestation, induction of labour (IOL) was started using prostaglandins and syntocinon IV, due to concerns associated with the fetus being small for gestational age (SGA). SGA may have been associated with the presence of an iliopsous abscess mass. However, the baby was in an oblique lie and not engaged into the maternal pelvis, also likely due to the presence of the iliopsous abscess mass. There was no cervical dilation and labour did not progress; an emergency caesarean section was performed due to concerns of fetal compromise related to the SGA baby and signs of fetal distress on the CTG during labour. General anaesthesia was recommended over spinal anaesthesia due to the presence paraspinal collection. The caesarean section was otherwise uneventful. A healthy baby of 2380 g was born, who did not need further resuscitation by the neonatal team. The placenta was sent to microbiology for further investigation for TB.

Referral was made to the trauma and orthopaeidc team. The pelvic and spine subspecialist teams were consulted regarding extensive tuberculosis osseous destruction and spinal-pelvic instability. A 2-stage procedure was urgently scheduled on 4th May 2017, with a primary anterior open reduction and internal fixation of the pelvis followed by a second stage posterior spinal instrumentation for spinal-pelvic dissociation pathological fractures.

The patient was positioned supine, and a urinary catheter was inserted to monitor the urinary output and for early detection of any bladder injury. A Stoppa approach was chosen using the previous Pfannenstiel incision from the caesarean section to access the pelvis.

With the help of reduction aids, closure of the sacroiliac diastasis was achieved. The reduction was primarily maintained with a 4-hole plate placed on the anterior aspect of the symphysis pubis followed by a 14-hole recon plate shielding the superior aspect of the symphysis pubis to strengthen the construct of fixation due to the poor bone quality. Adequate washout and closure of the wound in layers was achieved followed by well-padded dressing.

The second stage of the operation was led by the spinal team, changing the patient to a prone position. A posterior midline incision was performed. Paralumbar musculature was stripped and dissected from the spine to expose the facet joints, transverse processes, and posterior elements. Facet osteotomy of the inferior articular process was performed with ultrasonic bone cutters. Pedicle screws were inserted by the free hand technique with posterior spinal instrumentation from L4 to L5 connected to S2AI screws, thereby avoiding S1 screw fixation.

The lady was given co-amoxiclav as a prophylaxis against wound infection for a further 24 hours postoperatively. Antituberculous medications, as per microbiology team recommendations, were continued. She was admitted to neurosurgery ICU (NICU) for postoperative care and surveillance of neurology.

Physiotherapy was provided after surgery, allowing the patient to fully bear weight as pain tolerated, including a left-foot drop splint, to assist foot clearance for a more normal gait. The patient was able to sit and mobilise comfortably after the operation. Quadriceps static exercises and range of motion exercises were taught and given as part of her rehabilitation program.

### 4.1. Differential Diagnosis

TB is the great mimicker of disease. One of the differential diagnoses with a history of weight loss and osteolytic lesions seen on MRI were bone metastases. Therefore, tumour work-up was completed. Further investigations included a MRI of the spine and pelvis, bone biopsy, and paraspinal abscess drainage. The latter two showed no neoplastic cells in their samples.

Other differential diagnoses included abscess collection due to other pyogenic aetiology. This had been excluded with microbiology cultures and gram stains.

## 5. Outcome and Follow-Up

Over a 12-month period of follow-up with the infectious diseases team, the patient was able to complete her antituberculous pharmacotherapy according to their guidelines with no missed doses. No adverse effects were reported from the medication, and her phlebotomy, including full blood count and urea and electrolytes, were within the normal values. The patient was followed up in the pelvic and spinal trauma and orthopaedic outpatient clinic for the same period as well, and a rehabilitation program of physiotherapy was conducted three sessions per week at her home. This included walking short distances at home using an elbow crutch, a foot drop splint on the left leg, and full weight bearing as pain tolerated. Quadriceps strengthening exercises and range of motion exercises were also included. 4 weeks after discharge, the patient was able to do simple work activities. Climbing up the stairs was delayed until the patient's residual foot drop weakness had resolved. On 25/02/2018, the TB medication course was successfully completed.

**Figure 4:**
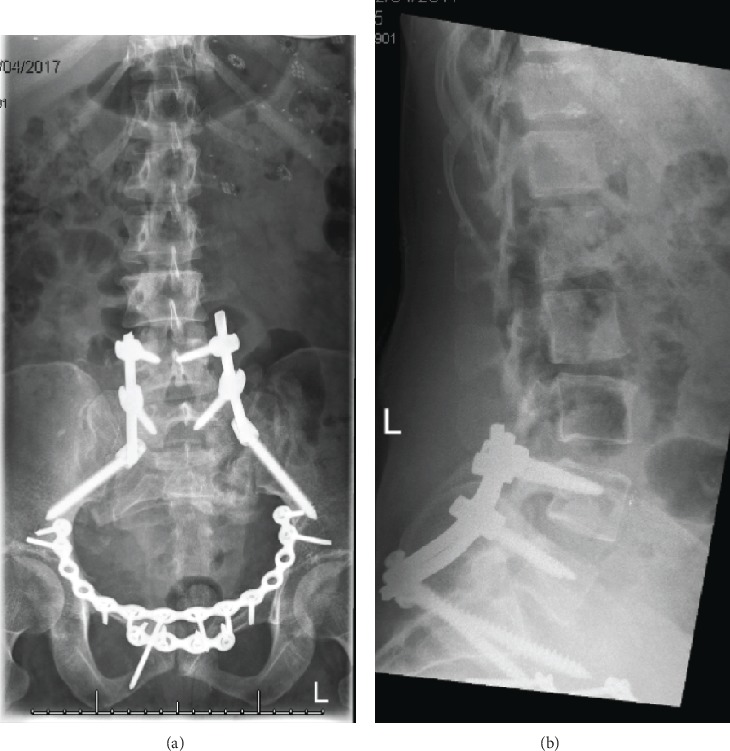
Postoperative pelvic X-rays and lumbosacral X-rays showing bone union in the pelvis and sufficient bone fusion in L4-S1 vertebrae in the antroposterior (a) and lateral views (b), respectively.

**Figure 5:**
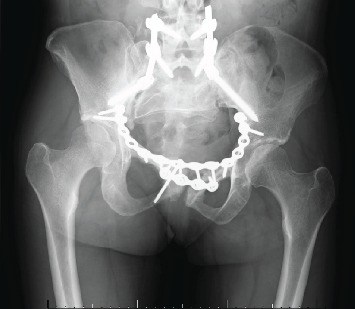
Failure of metalwork in one of the pelvic suprasymphyseal plates was identified on the left side and was managed nonoperatively.

A series of pelvic X-rays and lumbosacral X-rays were performed at 2 weeks, 6 weeks, 3 months, and 12 months postoperatively which showed ongoing bone union in the pelvis and satisfactory bone alignment (Figure 4). Failure of metalwork in one of the pelvic suprasymphyseal plates was identified and was managed nonoperatively (Figure 5).

**Figure 6:**
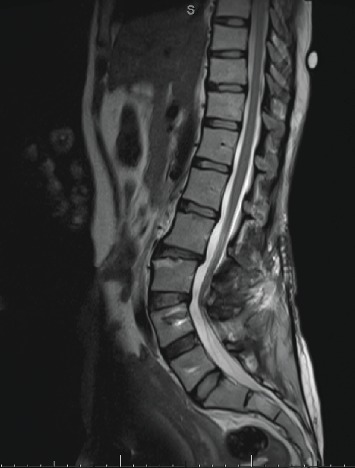
MRI was done 6 months postoperative fixation which revealed normal neurological strutures with no evidence of paraspinal cold abscess formation.

Foot drop resolved clinically with a minimal residual muscle weakness of the left leg; a dedicated lumbar spine MRI was done 6 months postoperative fixation with no evidence of paraspinal abscess formation or neuraxial derangement (Figure 6).

## 6. Discussion

Spinal tuberculosis is uncommon in the western world. Most of the patients with spinal tuberculosis in developed countries are immigrants from countries where tuberculosis is endemic. A review of data of musculoskeletal tuberculosis reviewed by Talbot et al. over a 6-year period was performed. From 1999 to 2004, there were 729 patients with tuberculosis. Approximately 8% (61) of the cases had musculoskeletal involvement; nearly 50% of these patients had spinal involvement. The majority (74%) of patients were immigrants from the Indian subcontinent [4].

Our report has suggested a successful collaboration of a multidisciplinary team, including the infectious disease team, orthopaedics, obstetricians, radiologists, microbiologists, and physiotherapists, in maintaining the safety of the child and stabilizing the patient. SFA and failure to progress led to an urgent caesarean section, obtaining samples from the placenta and leading to a culture-negative TB diagnosis. Due to the clinical picture and MDT collaboration, empirical anti-TB therapy was delivered appropriately. She finally went on to have stabilization for the spinal-pelvic dissociation secondary to destructive pathological tuberculous fracture.

The diagnosis of pulmonary TB is classically made with clinical, radiographic findings and a sputum smear test to detect the presence of acid-fast bacilli (AFB) [7]. In our case, the three tests were negative for TB, raising suspicions of a case of negative sputum smear and culture-negative TB. The diagnosis and decision of initiating anti-tuberculous therapy (ATT) was made when considering the extrapulmonary symptoms and musculoskeletal involvement. Antituberculosis therapy poses problems due to its side effect and potential teratogenic effect [8]. In our case, rifampcin, isoniazid INH, pyridoxine, and ethambutol were prescribed one day before delivery with no recorded side effects after the MDT decided it was best to proceed with empirical treatment.

The gold standard for the diagnosis of TB is a culture or nucleic acid amplification assay. The Xpert MTB/RIF assay is a PCR test that can identify both *Mycobacterium tuberculosis* (MTB) and rifampicin resistance, with cultures typically being the more sensitive method for the diagnosis [3, 9]; however, the results in our case showed no positive results adding to the challenges faced within this case study.

The presentation of tuberculosis in pregnant women is similar to that in nonpregnant women, but diagnosis may be delayed as early symptoms may be masked as they are related to physiological changes and weight gain in pregnancy [10]. Symptoms may include increased respiratory rate, loss of appetite, and fatigue. If there is no delay in diagnosis, pregnant women have the same outcomes as nonpregnant women. The presence of the suprasacral masses together with the neurological involvement in our case have been the key signs for raising the suspicion of spinal tuberculosis and initiation of TB chemotherapy. This was supported with the positive radiological findings on the urgent MRI scan done on admission despite any lesions seen on the chest X-ray.

Although rare in pregnancy in the UK (less than 1 : 2000), the incidence of tuberculosis is rising generally. Some small studies have found that tuberculosis in pregnancy has been associated with increased risks to infants, including prematurity, low birth weight (LBW), and small for gestational age (SGA) infants, as compared to infants of women without TB in keeping with our case. There are important differences in the epidemiology of TB in pregnancy compared with nonpregnant women due to the rarity of the event. It is accepted that a delay in TB diagnosis is not uncommon during pregnancy, but one must have a high level of suspicion with the appropriate background history [9]. In contrary, some studies have documented no significant difference in perinatal outcome between mothers with TB and unaffected mothers [8].

According to NICE guidelines, when there are clinical signs for the diagnosis of TB, treatment should be commenced without waiting for culture results [11]. WHO states that pulmonary and extra pulmonary TB diseases should be treated with the same regimen [12] as followed within this case study. Our surgical intervention was supported by NICE guidelines (2016), which recommend referring patients with spinal TB for surgery if there is spinal instability or evidence of spinal cord compression.

## 7. Conclusion

This is the first case report of surgical stabilization of a highly unstable spinal-pelvic fracture dissociation due to an extrapulmonary tuberculous spinopelvic destructive skeletal lesion in a gravid female. Imaging is important to delineate the fracture pattern, and MRI and CT both have a role, despite foetal radiation safety. Empirical ATT must be considered early as there is a high risk of a culture-negative TB diagnosis. Normal delivery can be attempted, but in our case, there was failure to proceed with SGA and an emergent caesarean section was performed safely.

Orthopaedic intervention was for gross instability, and an anterior Stoppa approach was used to access and stabilize the pelvic ring by open reduction and internal fixation. A second stage posterior approach was required to stabilize the spinopelvic dissociation using pedicle screws and iliac bolts. This case highlights the importance of maintaining a high level of suspicion for extrapulmonary tuberculosis for intractable back pain for a gravid female from the subcontinent.

## 8. Learning Points/Take Home Messages

1. A clinical picture could be a key factor in the diagnosis of tuberculosis even if the investigations were deemed negative
2. A clinical picture of tuberculosis could be challenging in pregnancy, as diagnosis can be delayed by the nonspecific nature of the symptoms which can be ascribed to pregnancy
3. The exact incidence and prevalence of spinal tuberculosis in most parts of the world are not known; however, it is seen primarily in immigrants coming from endemic countries, which should raise the index of suspicion during examination
4. Lumbopelvic tuberculosis complicated with neurological deficit in third-trimester pregnant women is considered a rare case, and there is not enough literature to review this case report
5. Sputum smear and culture-negative TB is considered a serious diagnostic challenge
